# Effects of cyclic loading on the shear bond strength of metal orthodontic brackets bonded to resin composite veneer surface using different conditioning protocols

**DOI:** 10.1186/2196-1042-14-14

**Published:** 2013-06-23

**Authors:** Shaza M Hammad, Mai S El Banna

**Affiliations:** Orthodontic Department, Faculty of Dentistry, Mansoura University, El Gomhoria Street, Mansoura, 35516 Egypt; Operative Dentistry, Faculty of Oral and Dental Medicine, Misr International University, Cairo, Egypt

**Keywords:** Cyclic loading, Bond strength, Orthodontic brackets, Laminate veneers

## Abstract

**Background:**

The aim of this research is to evaluate cyclic (CSBS) and static shear bond strengths (SSBS) of metal orthodontic brackets bonded to composite laminates using different conditioning protocols.

**Methods:**

A total of 80 direct nanofilled composite laminate veneers were prepared on permanent incisors and divided into four equal groups according to different surface treatments. In group 1, diamond bur was used. In group 2, microetcher (50-μm alumina particles) was utilized. In group 3, 38% phosphoric acid treatment for 60 s was done. In group 4 (control group), metal brackets were bonded to the untreated veneer surfaces using no-mix adhesive resin. SSBS testing was carried out for ten specimens, while CSBS testing was done for another ten specimens from each group. The data were subjected to analysis of variance and Scheffe post hoc test. The chi-square test was used to determine significant differences in the adhesive remnant index scores among different groups.

**Results:**

Statistically significant difference was only found between SSBS of brackets bonded when surface treatment was done using the diamond bur, microetcher, and the phosphoric acid at *P* < 0.05. With regard to CSBS, the use of bur treatment and microetching achieved the highest values; however, there was no significant difference between these two groups. With phosphoric acid, surface treatment achieved the lowest CSBS value; there was no significant difference between this group and the control group. The SSBS was significantly higher than CSBS in all groups.

**Conclusions:**

Roughening composite laminate veneers with either diamond bur or microetcher could be used successfully as an alternative to provide higher bond strength than phosphoric acid surface treatment. Cyclic loading significantly decreased bond strength.

## Background

Currently, an increasing number of adults are seeking orthodontic treatment, leading to an increased frequency of clinicians placing orthodontic appliances on teeth restored with resin composite restorations (RCRs) or resin laminate veneers [1–4]. This is associated with two major concerns. Firstly, the bond strength should be strong enough to withstand the forces applied during the orthodontic treatment. Secondly, the generated bond strength should not be too strong, thus leading to damage to the RCRs during debonding. The clinician needs to take care to preserve the integrity of the RCSs [5].

Several methods have been suggested to address this concern. These involve mechanical or chemical approaches to roughen the surface and increase the surface area for bonding. Mechanical preparation includes sandblasting [6–9] and surface grinding with carbide bur [10, 11] or diamond bur [6, 10]. Chemical methods include etching with orthophosphoric acid [6, 9, 10] and with hydrofluoric acid [7, 9, 11], and the application of a silane primer (porcelain primer) [9, 10, 12], dentine bonding agent [10], or plastic conditioner [6–9]. However, there is no agreement on a preferred protocol [13–16].

The type of composite resin may also affect the bond strength of orthodontic attachments [13]. Recently, nanofill composite resin has been introduced as a universal re\storative material. The strength and esthetic properties of nanocomposite allow the clinician to use it for both anterior and posterior restorations [17]. Unfortunately, bonding orthodontic attachments on nanofill composite resin may result in more frequent bond failure than bonding to other composite resins, as reported by previous studies [13, 18].

Brackets are subjected to cyclic stresses caused by mastication, occlusion, and orthodontic appliances [19–21]. Although these cyclic stresses could be of lower magnitude than the static bond strength of the bonded bracket, the repeated cyclic stresses occurring throughout the treatment period could lead to failure of the bracket, a condition referred as fatigue. Fatigue is the phenomenon through which failure is induced by subjecting the material or structure to repeated subcritical loads [22, 23]. Since it is very important to simulate the oral environment condition in the *in vitro* bond strength studies, it is advantageous to evaluate the effects of cyclic loading on bond strength. Therefore, the present study was conducted to evaluate and compare the cyclic shear bond strengths (CSBSs) and static shear bond strengths (SSBSs) of metallic orthodontic brackets bonded to resin laminate veneer surfaces (nanocomposite) using different conditioning protocols.

## Methods

A total number of 80 human permanent incisors, without caries, obvious defects, or attrition, extracted for periodontal causes over a period of 6 months, were selected for this study. The teeth were thoroughly cleaned and stored in distilled water and thymol at room temperature. The preparation of the labial surfaces of all the incisors and the direct application of the nanofilled composite (Tetric Evo-ceram, Ivoclar Vivadent AG, Schaan) laminate veneers were carried out by the same clinician according to the manufacturer’s instructions. All nanofilled composite laminate veneers were finished using diamond bur and aluminum oxide polishing paste. The 80 teeth were then divided into four groups of 20 teeth; each group was allocated to receive a different laminate surface treatment. In group 1, the laminates were roughened with a diamond bur with grit sizes of 125 to 150 mm (863 Grit; Drendell and Zweilling, Berlin, Germany). In group 2, the laminates were abraded with a microetcher (50-μm alumina particles; Microetcher ERC, Danville Engineering Inc., Danville, CA, USA). Group 3, the laminates were exposed to 38% phosphoric acid (3M ESPE, St Paul, Minnesota, USA) for 60 s, then rinsed with water for 60 s, and dried with compressed oil-free air. Group 4 is the control group which receives no surface treatment. Bracket bonding was carried out after 24 h of laminate placement.

### Bracket bonding

Stainless steel upper incisor brackets (Dentaurum GmbH & Co. KG, Ispringen, Germany) with a base area of 10.23 mm^2^ were then randomly bonded to the laminates’ surfaces with a no-mix adhesive resin (Granitec®, Confi-Dental Products Company, Louisville, CO, USA). A thin layer of adhesive primer was painted on the surface of each laminate. The adhesive resin paste was applied to the bracket base and the bracket seated on the surface of the laminate by the same clinician [5, 18]. Excess adhesive resin was removed with an explorer before polymerization with a curing light according to the manufacturer’s directions (from mesial and distal directions for 20 s; each direction for each bracket). To facilitate debonding, the teeth were mounted in acrylic resin blocks (Orthoresin, De Trey, Dentsply, Weybridge, UK) before the brackets were bonded. The buccal surfaces were close to parallel with a debonding blade. Each group of 20 specimens was further subdivided into two groups of ten. The specimens in each subgroup were then randomly subjected to stress tests. In subgroup 1, the specimens were exposed to static compressive load (SSBS), while in subgroup 2, the specimens were exposed to cyclic load to evaluate the CSBS.

### Testing procedures

The bond strengths of all specimens were determined using universal testing machine (Model LRX-Plus; Lloyd Instruments Ltd., Fareham, UK) with a load cell of 5 kN, and the data were recorded using computer software (Nexygen-MT; Lloyd Instruments).

### SSBS testing

Each specimen was mounted on the lower fixed component of the machine. The brackets were subjected to a compressive loading in the occlusogingival direction at a crosshead speed of 0.5 mm/min via monobeveled chisel attached to the upper movable component of the testing machine. The load was applied under the incisal wings at the base of each bonded bracket. The load required to dislodge each bracket was recorded in Newtons. The obtained values were converted into mega-Pascal units (MPa), according to the following equation: SBS = *F* / *A* (N/mm^2^ or MPa), where *F* is the debonding force in Newtons, and *A* is the cross-sectional surface area of the bracket base in square millimeters.

### CSBS testing

Each specimen was mounted in the testing machine (Model LRX-Plus; Lloyd Instruments Ltd.) with a loadcell of 5 kN, and the data were recorded using computer software (Nexygen-MT; Lloyd Instruments) as previously mentioned in the evaluation of SSBS. The specimens underwent cyclic loading by means of a monobeveled steel chisel that was attached to the upper movable component of the machine. The load was applied at the base of the bracket in the occlusogingival direction. The load profile was in the form of a wave at a rate of 1 Hz. Compressive shear fatigue test for 5,000 load cycles or until bond failure was determined by testing according to the staircase (up-and-down) method [24, 25]. The first specimen was tested at the approximate value of about 25% of the static shear bond strength previously evaluated [26]. Then, the load was raised by a fixed amount of 20 Newton (N) for the next tested specimen. On the other hand, if the bracket did not survive, i.e., the bond failed at some point during the 5,000 cycles, the load was lowered also by the same fixed amount of 20 N for the next specimen. This procedure of raising the maximum load by 20 N following a test where the orthodontic bond has not failed and lowering the load by the same fixed amount following a bond failure was continued for each following specimen until all ten specimens in each group were evaluated.

The mode of bond failure was assessed according to the amount of adhesive left on the laminate surfaces utilizing the adhesive remnant index (ARI) [27]. The ARI ranges from 0 (no adhesive left on the laminate surface) to 3 (all adhesive left on the laminate surface). Less than 50% of the adhesive left on the laminate surface yields a score of 1, while more than 50% of the adhesive left on the laminate surface yields a score of 2.

### Statistical analysis

Statistical analysis was performed using SPSS software, version 17.0 (Statistical Package for Social Sciences, SPSS Inc., Chicago, IL, USA). The means and SDs of SSBS and CSBS were calculated for all groups. The obtained data were subjected to analysis of variance and Scheffe post hoc tests to determine the significant differences among groups. The Student’s *t* test was used to determine the significant differences between the means of SSBS and CSBS. The chi-square test was used to determine the significant differences in the ARI scores among different groups. The significance for all statistical tests was predetermined at *P* < 0.05.

## Results

### SSBS results

Highest SSBS values were recorded with the brackets bonded to laminates treated with diamond bur followed with those treated with microetcher (6.44 ± 0.12 and 6.01 ± 0.02 MPa, respectively; Table 1).

**Table 1:** **Mean static and cyclic shear bond strengths, standard deviations, and results of the statistical tests**

Group	Means ^a^ and standard deviation (MPa)		***t*** Test ***P*** value (static vs. cyclic)	CSBS/SSBS (%)
	**Static shear bond strength**	**Cyclic shear bond strength**		
Diamond bur	6.44 ± 0.12 A	5.85 ± 0.03 A	>0.001	90.83
Microetcher	6.01 ± 0.02 B	5.31 ± 0.04 A	>0.001	88.35
Phosphoric acid	5.14 ± 0.03 C	3.25 ± 0.03 C	<0.001	63.22
Control	5.04 ± 0.05 C	3.84 ± 0.93 C	<0.001	76.19

^a^Means with the same letters in the same column are not significantly different at *P* < 0.05, according to the Scheffe test.

Lowest SSBS results were recorded with the brackets bonded in the control group (5.04 ± 0.05 MPa) followed by the group treated with phosphoric acid (5.14 ± 0.03 MPa, with no statistical significant difference between the two groups.

### CSBS results

Highest CSBS values were also recorded with the brackets bonded to laminates treated with diamond bur followed with those treated with microetcher (5.85 ± 0.03 and 5.31 ± 0.04 MPa, respectively), with no statistical significant difference between them. On the other hand, the lowest CSBS values were recorded for the CSBS of the brackets bonded to laminates treated with phosphoric acid and then the control group (3.25 ± 0.03 and 3.84 ± 0.93, MPa respectively). Both groups showed a statistically significant difference with the CSBS of brackets bonded to laminates treated with diamond bur and those treated with microetcher (*P* < 0.05).

Results also revealed that the mean CBSS of the brackets bonded to laminates treated with diamond bur was 90.8% from their SSBS mean. Next were the brackets bonded to laminates treated with microetcher, as the mean of CSBS was 88.35% of their SSBS mean. The CBSS to the SSBS means for the control group and the brackets bonded to laminates treated with phosphoric acid were 76.19% and 63.22%, respectively.

The highest ARI score in all groups was 0 (no adhesive left on the laminate surface; Table 2).

**Table 2:** **Frequency distribution of the ARI scores for the test and control groups** ^**a**^

Group	Static shear bond strength score				Cyclic shear bond strength score			
	**0**	**1**	**2**	**3**	**0**	**1**	**2**	**3**
Diamond bur	5	3	2	0	4	3	1	0
Microetcher	5	2	3	0	3	2	1	0
Phosphoric acid	7	2	1	0	6	2	0	0
Control	8	2	0	0	7	1	0	0

^a^ *x* ^2^ = 4.55, *x* ^2^ = 1.87, *P* = 0.60, *P* = 0.92.

## Discussion

There are many factors that potentially influence the bond strength of orthodontic attachments to composite resin surfaces such as the type of composite resin, the film thickness of adhesive resin, moisture, contamination, the dimension and geometry of the bracket base, storage conditions, aging of the composite, and method of testing [5–8]. Surface treatment techniques are another crucial factor influencing the bond strength values. These involve mechanical or chemical approaches to roughening the surface and increasing the surface area for bonding. Inappropriate surface treatment of laminate veneers before bonding orthodontic brackets can result in fracture or loss of the underlying laminate during debonding; furthermore, adhesive remnants require removal [14]. The additional cost of cleanup or replacement of the restoration must be considered. Additionally, in an *in vivo* situation, bonding systems are more likely to be challenged by repeated applications of stresses that are below the maximum stress that these systems could withstand. Therefore, fatigue test results would provide more accurate predictions of the *in vivo* performance of orthodontic bonding systems [11, 19–28].

The present study was conducted to evaluate the bond strengths of metal orthodontic brackets bonded to resin laminate veneer surfaces (nanocomposite) using diamond bur, microetcher, or phosphoric acid surface treatments. Since it is advantageous to simulate the oral environment condition in the *in vitro* bond strength studies, both CSBSs and SSBSs were evaluated. The clinical situation was simulated by preparing standardized restorations in extracted human incisors.

The highest SSBS of metal brackets bonded on laminate surfaces treated with diamond bur could be attributed to the fact that the mechanical abrasive methods which increase mechanical interlocking are perhaps the most significant factor contributing to bond strength, increasing the surface roughness more than did etching with phosphoric acid, which subsequently improved the mechanical retention of the adhesives [5, 9, 18]. This can be explained by the formation of deep craters and streaks which are sufficient for micromechanical retention of orthodontic adhesives. Other studies have reported that sandblasting with alumina particles (microetcher) creates the highest bond strength for both repaired hybrid and nanofill resin composite [20, 29, 30]. On the other hand, orthophosphoric acid etching could not change surface topography of a nanofill composite resin. As phosphoric acid dissolves the inorganic component of the enamel prism, it does not affect the organic component. Consequently, one would not expect the phosphoric acid to have any effect on the composite surface except for enhanced cleaning of the composite resin surface [3–6, 20].

CSBS provides more realistic and valuable information (compared to SSBS) about the material’s long-term performance in the clinical situation. In other words, CSBS could be used as a more reliable predictive indicator of bond strength in the oral environment [20–23]. In the present study, the staircase method was used to determine CSBS. In this method, the data are concentrated around the mean stress; hence, the number of specimens is smaller than that required with other techniques. Subsequently, this method is less time consuming [31]. In spite of the relatively low magnitude of the cyclic loads, this method could lead to microcracks and structural failure, a phenomena commonly known as fatigue [23, 31]. Several factors could affect fatigue, such as stress concentration, corrosion, temperature, overload, microstructure, and residual stresses [19]. In the current study, the utilization of either surface bur treatment or microetching provided comparable CSBS values. Accordingly, the bonded brackets could withstand the stresses of mastication and orthodontic appliances (fatigue resistance) at the same level.

In addition, the results of the present study revealed that CSBS values were significantly lower than the SSBS values (*P* < 0.05). The use of percentages clearly indicates that cyclic fatigue tests produce results that are much lower than their static counterpart and facilitates comparison with previous reported studies. This finding was in harmony with those of other studies [20, 32, 33]. This could explain why bracket failure occurs in the oral environment when teeth are subjected to forces of lower magnitude compared to their respective static magnitude. Other studies [20, 30] reported even lower ratios when comparing cyclic to static loading. They reported ratios of 51% for their tests of primed composite resin adhesion and 60% for compressive strengths of porcelain bars. They attributed that decrease to mechanical and masticatory stresses affecting the bonds in the oral environment in addition to other factors, such as moisture contamination during bonding, intraoral thermal fluctuation, and the constant bathing effects of saliva. Therefore, it is advantageous to evaluate the effect of cyclic loading on the bond strength of the adhesive systems utilized in orthodontic practice.

The bond strength between an orthodontic bracket and a laminate veneer should be sufficient to withstand the forces generated by mastication, last the duration of orthodontic treatment but allow straightforward removal at the end of treatment without damage to the underlying restoration. The minimum bond strength for orthodontic purposes falls within the range of 6 to 8 MPa [19]. It has also been reported that the minimum bond strength needed by a stainless steel bracket to be clinically acceptable is 2.86 MPa [32]. In the present study, bond strengths for brackets bonded to composite laminate surfaces pre-treated with both diamond bur and microetcher lie approximately within this acceptable range, being higher than the former. However, significantly high bond strength between the adhesive resin and a restoration has disadvantages. Fracture or loss of the underlying restoration during debonding can occur. Additionally, sandblasting with aluminum oxide particles (microetcher) may be safer than utilizing burs or stones, since the procedure is more uniform and less aggressive [33].

The ARI scores showed that bond failure predominantly occurred between the veneer and the adhesive as the majority of the adhesive remained on the bracket bases in all groups. Further research is still needed, and as with any *in vitro* study, caution must be used when attempting to extrapolate these results to a clinical setting.

## Conclusions

Preparing composite laminate surfaces with both a diamond bur and a microetcher showed clinically accepted SSBS and CSBS for bonded stainless steel brackets. Mechanical surface treatments achieved statistically significant higher SSBS and CSBS values compared to the group treated with phosphoric acid and the control group. Cyclic loading significantly decreased bond strength.
